# An activity-friendly environment from the adolescent perspective: a concept mapping study

**DOI:** 10.1186/s12966-018-0733-x

**Published:** 2018-10-16

**Authors:** L. M. Hidding, M. J. M. Chinapaw, T. M. Altenburg

**Affiliations:** Department of Public and Occupational Health, Amsterdam Public Health Research Institute, Amsterdam UMC, Vrije Universiteit Amsterdam, Van der Boechorststraat 7, NL-1081 BT Amsterdam, The Netherlands

**Keywords:** Youth, Concept mapping, Physical activity, Environment, Activity friendliness

## Abstract

**Background:**

In today’s society, few adolescents meet physical activity guidelines and effects of physical activity promoting programmes are disappointing. In studies exploring determinants of physical activity, the perspective of adolescents themselves is largely lacking. Also, there is a lack of knowledge on potential environmental determinants of adolescent physical activity. Therefore, this study aimed to explore adolescents’ perspectives on characteristics of an activity-friendly environment.

**Methods:**

Concept mapping meetings were conducted with four secondary school classes, including 115 adolescents (13–17 years). Each student generated ideas regarding the characteristics of an activity-friendly environment. For each school class, ideas were combined and identical ideas were removed. Next, students individually sorted all ideas, based on self-perceived similarity, and rated their importance on a five-point Likert-scale. A concept map was created for each school class using multidimensional scaling and hierarchical cluster analysis. Finally, the researchers named the potential environmental determinants within the clusters.

**Results:**

The concept maps depicted 23 unique potential determinants of activity friendliness, of which 15 were similar across all school classes. Potential determinants were categorized in the physical-, social-, economic-, and motivational domain. The most frequent and important adolescent-perceived determinants of activity friendliness across all school classes belonged to the physical domain, e.g. a suitable area including a proper surface for a variety of sports, and good lighting in the playground.

**Conclusions:**

Our findings show that adolescents perceive potential determinants in the physical and economic domain as most important for activity friendliness, indicating that future interventions might benefit from targeting potential determinants within these domains.

**Electronic supplementary material:**

The online version of this article (10.1186/s12966-018-0733-x) contains supplementary material, which is available to authorized users.

## Background

Globally, many adolescents do not meet the recommendations for physical activity. In 2012, only 20% of 13- to 15-year-old adolescents engaged in at least 60 min of moderate to vigorous physical activity per day, according to self-reports [1]. Importantly, a large body of evidence exists for the beneficial effects of physical activity of at least moderate intensity on both physical and mental health [2, 3]. Furthermore, physical activity tracks into adulthood [4–7], emphasizing the need for promoting healthy habits from an early age.

Adolescents’ physical activity is affected by multiple determinants that are frequently categorized in the following domains: 1) demographic and biological; 2) psychological, cognitive and emotional; 3) behavioural; 4) social and cultural; and 5) physical environmental [8–10]. Multiple reviews have identified correlates of adolescents’ activity behaviour across these domains. However, evidence was often insufficient or inconsistent due to a lack of high-quality studies, especially for physical and social-cultural environmental correlates as few studies focused on these domains [8–10].

Nevertheless, interventions addressing children’s and adolescents’ physical and social environments have shown promising effects on physical activity behaviour [11–13]. However, these interventions were predominantly limited to playgrounds and public parks disregarding adolescents’ environments in general, e.g. not including the route to school or commonly used hangouts. Furthermore, a systematic review on the effectiveness of physical activity interventions in youth found inconclusive evidence for the effectiveness of the included environmental interventions [14]. The majority of these environmental interventions were limited to the school environment, indicating the lack of focus on the environment in general.

As adolescents are experts of their own behaviour, their perspectives could bring new and important insights [15] that may enhance the effectiveness of physical activity interventions. Adolescents’ perspectives on potential environmental determinants have previously been examined in qualitative research [16, 17]. However, these studies generally focused on specific places in adolescents’ neighbourhoods, and the barriers and facilitators of physical activity at these specific places. Our study focuses on the environment in general.

Therefore, the aim of this study was to broadly explore adolescents’ perspectives on determinants of an activity-friendly environment, by performing concept mapping meetings with 13- to 17-year-old students attending secondary school. Concept mapping incorporates the participants’ perspectives throughout the process [18] by giving them the opportunity to share their ideas and opinions. Furthermore, we aim to indicate which potential determinants are most relevant to adolescents by letting the adolescents rate the importance of all identified potential determinants.

## Methods

### Participants

Participants were recruited through purposive sampling, between November 2015 and December 2016, aiming to include students attending different educational levels of secondary school aged between 13 to 17 years old. Schools were contacted through a personal network and were approached based on differences in socio-economic status and location, i.e. schools located in a village and a city. The socio-economic status of the participating schools was obtained using the status-scores document from the Dutch Social and Cultural Planning Agency and the postal codes of the school. Socio-economic status was divided in tertiles, i.e. low, medium, and high socio-economic status. Four secondary school classes across three schools located in the surroundings of Amsterdam were invited to participate (one in a village, two in cities). All schools/classes agreed to participate (response rate classes and schools 100%): one year 2 and one year 4 of 4-year Dutch pre-vocational secondary education (comparable to year 9 and 11 in UK secondary education), and one year 2 and one year 4 of 6-year Dutch pre-university secondary education. In Dutch schools the pre-vocational level prepares students for vocational college, and takes 4 years. The pre-university level prepares the students for university, this level takes 6 years. Students were 13- to 17 years old, and class sizes ranged from 20 to 46 students. All students in the participating classes were eligible for participation. Information letters and informed consent forms were sent to the students and their parents.

The VU University Medical Ethical Committee approved the study protocol. No identifying participant information was collected for the purpose of this study, and written informed consent was signed by one parent and the participating student.

### Study design

Data collection took place between December 2015 and January 2017. For this study the concept mapping method was used, a mixed method including a qualitative data collection, and a quantitative data analysis. Several successive steps were performed: idea generation towards a seeding statement; sorting and rating of the generated ideas; statistical analysis; and interpretation of the concept maps [18, 19]. Study size was determined by data saturation. Students participated in two steps in separate sessions: in the first session, statements were generated and in the second session statements were sorted and rated. For practical reasons we were not able to organize parallel sessions for all classes, therefore one concept map for each class was generated.

At the start of the concept mapping sessions, students’ age and gender were registered. The detailed concept mapping procedure is presented below.

### Procedures

#### First session

For the first session, each school class was divided into smaller groups (*n* = 7 to 12 students per group), resulting in 12 concept mapping sessions (2 to 4 groups per class). Sessions were held during school hours, at school (one school, two classes) and at the Amsterdam UMC (two schools, one class each). The sessions took approximately one hour and were facilitated by two researchers. The session started with two warm-up questions to stimulate students’ understanding of environments that positively or negatively influence their activity behaviour: ‘In which locations or environments are you quite physically active?’, and ‘In which locations or environments are you quite inactive?’. Next, the seeding statement was presented, formulated both in a question and a sentence to give the students the opportunity to choose the statement they found easiest to answer:

*‘When do you think of an environment as being activity-friendly?’*

*‘I think an environment is activity-friendly when it is/has…’*

An individual brainstorm took place during which the students generated as many ideas as they could think of to answer the seeding statement. After the individual brainstorm, the students shared their ideas, one by one, in a group brainstorm, resulting in a list of unique ideas.

In preparation for the second session, researchers combined the ideas generated in the smaller groups during the first session for each school class. Identical ideas were removed or combined resulting in a list of unique ideas for each school class, a maximum of 98 ideas per school class was maintained due to settings of the used concept mapping software ‘Ariadne’. In the case of disagreements, discussions took place (LH and TA) until consensus was reached.

#### Second session

The second concept mapping sessions were held at school during school hours, approximately one week after the first session, and included the same students as the first session. During the second session, students sorted and rated the ideas using a personal page in the online programme. The students sorted the ideas in different piles based on similarities between ideas. A ‘miscellaneous’ pile was not allowed. The software allowed a minimum of three piles and a maximum of ten. After the sorting task, the students named the different piles, with a title covering the underlying ideas. Next, students rated all ideas on a 5-point Likert-scale ranging from: 1) really unimportant to 5) really important.

#### Statistical analysis

Statistical analyses were performed using the software programme Ariadne [20], which identifies patterns and visualizes these patterns in a concept map, using multidimensional scaling and hierarchical cluster analysis. A concept map for every school class was made, resulting in four concept maps (see Additional file 1). Based on the sorting by the individual participants, the different ideas were arranged on the concept map. Ideas that were sorted together more often by the participants in the sorting task were placed closer to one another on the map. Furthermore, by default the programme creates eight clusters, ideas close to each other on the map appear together in a cluster. In addition, the average importance rating of each idea is calculated based on the individual participants’ ratings. The cluster compositions and average importance ratings of the underlying ideas are shown in Additional file 2.

#### Interpretation

Two independent researchers (LH and TA) adapted the final number of clusters per concept map by interpreting the underlying ideas in each of the generated clusters, aiming for each cluster to represent a clear topic, or multiple clear topics. In short, to optimally represent students’ ideas, the researchers critically reviewed all ideas within a cluster, and checked whether combining or splitting up clusters gave a better representation of the underlying ideas. After defining the final number of clusters, the clusters were named by taking into account the titles given by the students during the sorting task. As statistical significance is not always the best indication for representing qualitative data, some of the ideas within the computer-generated clusters were moved to existing or new clusters, as they fitted better with another topic. The majority of clusters represented multiple topics, i.e. potential determinants. To be able to compare similar potential determinants mentioned across schools, we refer to potential determinants instead of clusters in the remainder of the manuscript. Subsequently, the average importance of each potential determinant was calculated, based on the average importance ratings of the underlying ideas.

## Results

### Participants

A total of 115 students (37% girls; 14.2 ± 1.2 years old) participated in this study (response rate students 86%) and were included in the analysis. Forty-four students were from the 2nd year of 4-year pre-vocational secondary education (96% response rate), 14 from the 4th year of pre-vocational secondary education (70% response rate), 17 from the 2nd year of 6-year pre-university secondary education (96% response rate), and 40 from the 4th year of pre-university secondary education (100% response rate). The socio-economic status of the schools was in the lowest tertile for two of the schools, and in the highest tertile for the other school.

### Concept maps

Students generated between 61 and 98 unique ideas. The number of clusters in the final concept maps, i.e. researcher-adapted maps, ranged from seven to 12. As the majority of clusters represented more than one potential determinant, they were separated to provide a clear overview of similarities across classes (Table 1). For clarity reasons, the potential determinants were categorized in four domains (i.e. physical, social, economic, and motivational characteristics).

**Table 1:** Average importance rating^a^ of adolescent-identified determinants of an activity-friendly environment

Potential determinants	6-year pre-university secondary education		4-year pre-vocational secondary education		Average importance of all classes
	2nd year	4th year	2nd year	4th year	
Physical characteristics					
1. Clean	**4.2**	**3.2**	**3.9**	**3.5**	**3.7**
2. Attractive	**3.6**	2.6	**3.1**	**3.4**	**3.2**
3. Proximity	**3.3**	**3.3**	**3.3**	**3.4**	**3.3**
4. Attributes	**3.3**	**3.2**	**3.4**	**4.0**	**3.5**
5. Facilities	**3.4**	**3.2**	3.0	**4.1**	**3.4**
6. Well maintained	**4.4**	**4.0**	**3.4**	**4.5**	**4.1**
7. Suitable area	**3.1**	**3.4**	**3.3**	**4.2**	**3.5**
8. Weather	**3.4**	2.8	**3.1**	**4.2**	**3.4**
9. Safety	**3.8**	**3.5**	2.8	**4.0**	**3.5**
10. Variation	**3.1**	**3.3**	**3.1**	2.6	3.0
Social characteristics					
1. Presence of others (positive/negative)	2.8	2.8	2.8	**4.0**	**3.1**
2. Different target groups	NA	2.9	**3.1**	**3.5**	**3.2**
3. Ambience	**3.8**	**3.8**	2.5	**4.1**	**3.6**
4. Being allowed to be active	**3.3**	**3.8**	**3.7**	**3.1**	**3.5**
5. It is the norm to be active	NA	2.5	NA	NA	2.5
Economic characteristics					
1. Affordable	**3.4**	**3.6**	**3.7**	**3.1**	**3.5**
Motivational characteristics					
1. Challenging, motivating, exciting and adventurous	2.4	2.9	**3.2**	**3.4**	3.0
2. Rewards	1.8	**3.1**	2.6	NA	2.5
3. Seated activities not encouraged	2.2	2.0	2.6	NA	2.3
4. Distraction (positive/negative)	2.3	2.4	**3.2**	3.0	2.8
5. Organized activities	2.5	NA	2.7	NA	2.6
6. Active games	2.1	NA	**3.2**	NA	2.7
7. Being forced to be active	NA	NA	2.4	2.7	2.6

*NA* Not applicable, indicates a potential determinant was not indicated during the concept mapping session within this class; bold indicates the potential determinant is important

^a^Rated on a 5-point Likert-scale with higher scores indicating higher importance of the adolescent-perceived determinants

Within the physical domain, students from all classes mentioned the following potential determinants: 1) clean, e.g. ‘If the area is clean (no waste/dirt/sweat on the equipment)’; 2) attractive, e.g. ‘If there is a lot of green (nature) in the area’; 3) proximity, e.g. ‘If it is nearby and easily accessible’; 4) attributes, e.g. ‘Attributes available to be active with, like a parkour track, climbing rack, basketball net, or a trampoline’; 5) facilities, e.g. ‘If there are drinking water fountains in the area’; 6) well maintained, e.g. ‘If it is well-kept (no lawn without grass/broken climbing frames)’; 7) suitable area, e.g. ‘If there is space you can be active in and perform sports, a spacious/big sports field^’^; 8) weather, e.g. ‘If there is a possibility to go inside (if it starts raining or when it’s cold)’; 9) safety, e.g. ‘If you feel safe, if there are no groups of youths loitering for example’ and e.g. ‘If attributes (e.g. a  climbing frame) are made from strong material’; and 10) variation, e.g. ‘If there is variation in play equipment and quantities (enough to do for a longer period)’.

Within the social domain, the following potential determinants were mentioned by students from all classes: 1) presence of others (positive/negative), e.g. ‘If there is enough privacy, no non-sporting people watching others participating in a sport (if you are sweating/have a red face)’, and ‘If it encourages you to be active together’; 2) ambience, e.g. ‘If the ambience is nice’; 3) Being allowed to be active, e.g. If I’m allowed to be active (e.g. in school).

One potential determinant within the economic domain was found and mentioned by students of all classes: affordable, e.g. ‘If it is for free or cheap, for example, less expensive sports clubs for example’.

Within the motivational domain, two potential determinants were mentioned by students from all classes: 1) challenging, motivating, exciting and adventurous, e.g. ‘If you are physically challenged (enough weights for bench press, to be able to improve step by step)’ and ‘If there is a game element’; and 2) distraction (positive/negative), e.g. ‘If there is music in the background (e.g. for distraction)’ and ‘If there are no things around that distract you (a computer for example)’.

Table 1 shows the average importance of the potential determinants. Potential determinants that were rated important, i.e. average rating > 3, according to all classes were: 1) clean; 2) proximity; 3) attributes; 4) well maintained; 5) suitable area; 6) being allowed to be active; and 7) affordable. When looking at the overall average importance scores across all classes, the top three potential determinants rated as most important were: 1) well maintained; 2) clean; and 3) ambience.

## Discussion

This concept mapping study demonstrates that adolescents identified unique potential determinants of an activity-friendly environment belonging to four domains, i.e. physical, social, economic, and motivational characteristics. Potential determinants belonging to the physical, and economic domains were rated as most important across all four school classes. Within the social, and motivational domain, a number of potential determinants were also rated as important, although, this varied across school classes.

### Physical characteristics

According to all classes, the most important physical characteristics of an activity-friendly physical environment were: clean, well maintained, and proximity. Furthermore, the environment should have a variety of attributes available, such as play or sports equipment. Additionally, the suitability of the area was rated as an important aspect, for example there should be a proper surface for various kinds of sports/activities, good lighting, enough space, and a comfortable temperature. Our findings are in line with a systematic review including both qualitative and quantitative studies. The qualitative results found that adolescents perceived a clean and well maintained public open space as a facilitator for visiting public open spaces and engaging in physical activity, whereas a lack of cleanliness was perceived as a barrier. However, the few quantitative studies that addressed this topic, and were only focused on maintenance, not cleanliness, did not confirm this [16]. In contrast to our findings, a systematic review including quantitative studies found no evidence for an association between accessibility and proximity to recreation facilities and self-reported physical activity in adolescents [21]. On the other hand, low proximity (distance from home and lack of transportation to physical activity opportunities) was mentioned as a barrier for engaging in physical activity by adolescents in multiple qualitative studies [17, 22]. Similar to previous qualitative findings [16, 17], we found that the availability of a variety of attributes/equipment is an important characteristic of an activity-friendly environment, whereas having to bring additional equipment to a sports facility is perceived as a barrier for physical activity. Our finding is also in line with three systematic reviews that demonstrated a positive association of the availability of recreation facilities/equipment and opportunities for physical activity with adolescents’ physical activity [8, 23, 24].

Interestingly, the potential determinant ‘suitable area’, includes many detailed aspects of an activity-friendly physical environment for adolescents, such as proper lighting, surface, and temperature. Such details were generally not included in previous quantitative studies examining the association between physical environmental characteristics and youth’s physical activity [21, 25, 26]. However, previous qualitative studies reported more detailed aspects, adolescents mentioned that flat surfaces are required for open spaces, and sports opportunities with good lighting are perceived as a stimulus for engaging in physical activity [16]. In short, the importance of the physical environment seems well understood in the current literature. However, previous quantitative studies have primarily focused on a more global picture of the built environment such as the availability of a playground (yes/no), or proximity of a playground. Our study highlights the importance of focusing on more detailed aspects, such as maintenance of play equipment, a proper surface for a variety of sports, and good lighting in the playground.

### Social characteristics

In the social domain, ‘being allowed to be active’ was rated important by all classes. In addition, ‘ambience’ may be another important potential determinant, as it was rated important by three of the four classes. According to the students, important aspects of a good ambience in the environment include that it should be cosy together with others, and fun for everyone. Similarly, a systematic review of qualitative studies concluded that having fun was frequently mentioned as a physical activity facilitator in adolescents [17]. The importance of being together with others was also previously found to be important for adolescents’ physical activity, in both qualitative and quantitative research [22, 27, 28], although the main focus was on social support rather than having fun and cosiness.

The potential determinant ‘being allowed to be active’, included statements about the absence of strict rules or persons who restrict you in being active, ‘If you are free to do whatever you want to do (no one tells you what to do)’. The importance of ‘being allowed to be active’ is in line with findings of a systematic review of qualitative studies, stating that any strict rules or restrictions make public open spaces less attractive for adolescents for engaging in physical activity [16].

### Economic characteristics

All school classes perceived the affordability of opportunities for physical activity as an important potential economic determinant: opportunities for physical activity should be free or inexpensive, especially fun activities that are generally more expensive like laser games, and exercising at sports clubs. Likewise, a systematic review including qualitative studies stated that cost-related access problems to physical activity programmes was one of the reasons for adolescents not to participate in physical activity [17]. Furthermore, a quantitative systematic review found positive effects on physical activity when children were provided with free play equipment [24], however, this was found in younger children aged 6–12 years. In addition, a systematic review of reviews concluded that family income and socio-economic status were positively associated with adolescents’ physical activity [27], suggesting that costs of physical activity might hamper adolescents in being physically active. Though students from both educational levels within our study indicated the importance of affordability of physical activity opportunities, especially adolescents growing up in families with lower incomes and/or socio-economic status might benefit from more affordable physical activity opportunities [27].

### Motivational characteristics

Though the students indicated quite a few potential motivational determinants of the activity friendliness of the environment, none of the potential determinants was rated as important across all classes. However, a ‘challenging, motivating, exciting and adventurous’ environment was rated as important by both pre-vocational level classes. Students indicated that a challenging, motivating, exciting and adventurous environment includes for example original play equipment, activities or sports that you do not often engage in, the possibility to compete, the ability to keep track of your improvements, a game element, and a motivating external person, a trainer. Similarly, two previous quantitative studies examining adolescents’ ratings of different park features, demonstrated that adolescents perceived adventurous play equipment/playgrounds as important stimuli for being active in the park [29, 30]. The participants in the study of Hohepa et al. [22] also indicated motivational assistance, such as setting goals, and the use of a pedometer, as an activity-enhancing strategy. Furthermore, a qualitative study among 9- to 12-year-old children concluded that a challenging playground with the right degree of difficulty is important for fun active play [31].

### Future recommendations

Many adolescents do not meet physical activity guidelines and existing physical activity interventions have limited effects [1, 8–10]. Environmental interventions aiming to enhance adolescents’ physical activity show promising effects [11–13] and knowledge of potential environmental determinants and their relevance from the adolescent perspective found in this study may further improve such interventions. Future intervention studies should explore whether environmental interventions targeting the adolescent-perceived determinants can improve adolescents’ physical activity and identify which determinants are most important.

### Strengths and limitations

The focus on the adolescent perspective is a major strength of this study, as adolescents are experts of their own behaviour, and therefore can best indicate their needs. The broad focus on activity friendliness of all environments relevant to adolescents further strengthens our study. Current intervention studies aiming to improve physical activity mainly focus on a specific environment such as a nearby playground, whereas a broader perspective may be required to significantly influence adolescent’s physical activity. In addition, the high response rate, the broad age range, the considerable sample size, the variety in educational levels, and including schools in both urban and more rural areas are important strengths of this study, as it increases the representativeness of our findings for secondary school students in the Netherlands. In addition, during the last concept mapping session, no new potential determinants emerged, indicating saturation had been reached. This study might be limited by the fact that we were not able to compute one concept map for all school classes together. However, the four maps allowed us to identify the differences in ideas and main topics that were discussed during the idea generation between school classes. Another limitation might be that some of the students seemed very sports-orientated during the statement generation, while the aim of the study was to focus on physical activity in general. Lastly, all high schools were located in a more urban part of the Netherlands, inclusion of schools in more rural areas might have resulted in additional potential determinants.

## Conclusions

This study provides insight into unique adolescent-perceived determinants of an activity-friendly environment. Interestingly, our focus on environments in general, rather than focusing on an existing playground or specific neighbourhood, resulted in more – as well as more detailed – potential determinants of an activity-friendly environment. The most important adolescent-perceived determinants related to the physical and economic domains. However, interventions are needed to confirm that changing the adolescent-perceived determinants do indeed promote their physical activity levels.

## Additional files

Additional file 1: Concept maps. Four concept maps, one for each school class. (DOCX 372 kb)

Additional file 2: Cluster compositions and importance ratings. Cluster compositions and average importance ratings of the underlying ideas. (DOCX 45 kb)

## Abbreviations

LH: Lisan Hidding
MC: Mai Chinapaw
TA: Teatske Altenburg
