# Supplementary material for: An activity-friendly environment from the adolescent perspective: a concept mapping study
Source: Int J Behav Nutr Phys Act. 2018 Oct 16;15:99. doi: 10.1186/s12966-018-0733-x (PMC6192111; doi:10.1186/s12966-018-0733-x)
Supplement: Supplementary file 1 — Concept maps. Four concept maps, one for each school class. (DOCX 372 kb) [file 12966_2018_733_MOESM1_ESM.docx]

Additional file 1: Concept maps


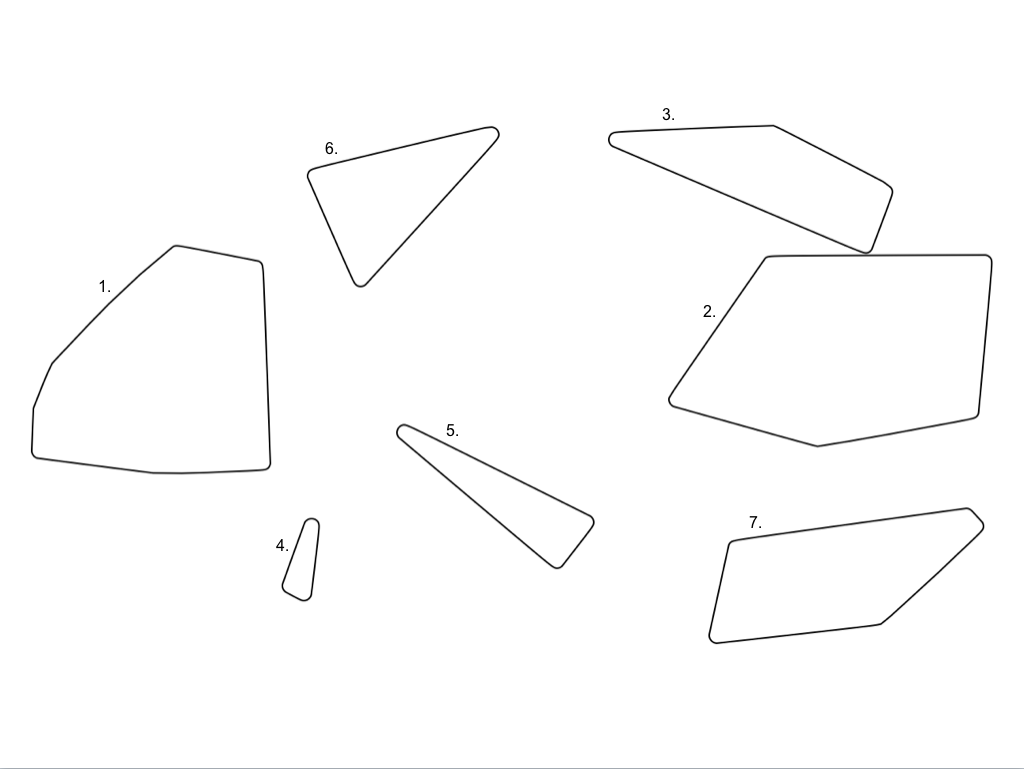


**Figure 1.** Concept map of year 2 of 6-year pre-university secondary education

Note that clusters closer together on the map have a stronger relation than clusters further away from each other;

Cluster 1: Variation and attributes; Cluster 2: Clean, well maintained, safe, and distraction (positive/negative); Cluster 3: Attractive and suitable area; Cluster 4: Rewards and organized activities; Cluster 5: Active games; Cluster 6: Facilities; Cluster 7: Ambience and presence of others (positive/negative);

Note that two new clusters (not shown on the map) were created as a result of reallocation of statements: Cluster 8: Affordable and proximity; Cluster 9: Challenging, motivating, exciting, and adventurous.


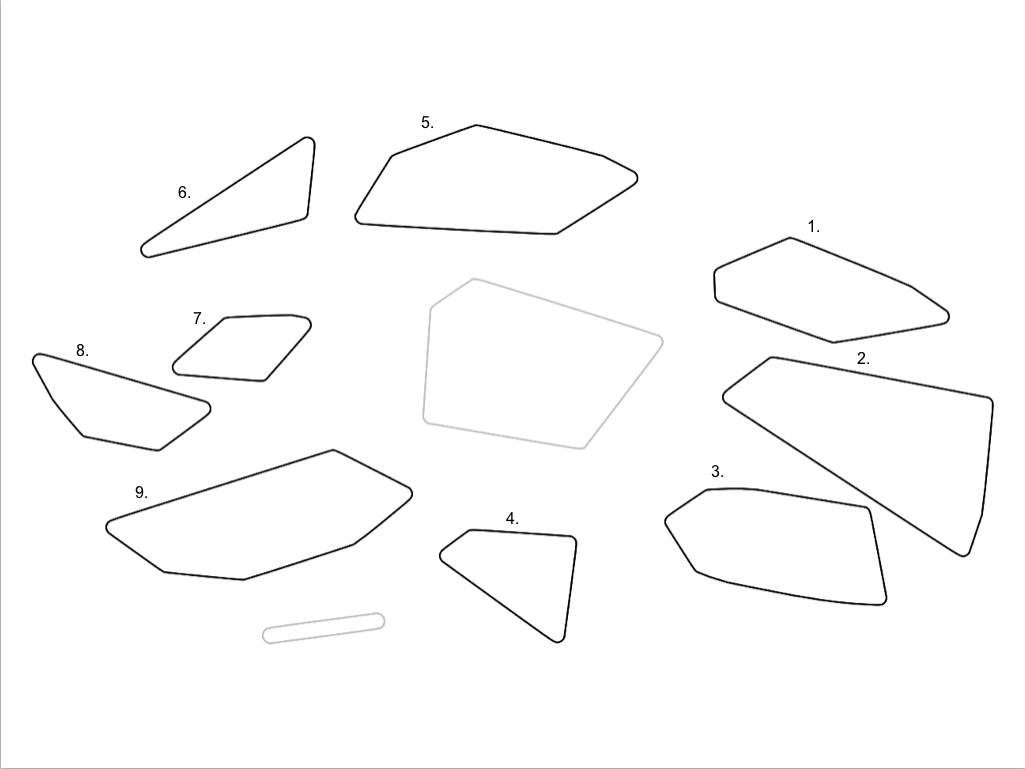


**Figure 2.** Concept map of year 2 of 4-year pre-vocational secondary education

Note that clusters closer together on the map have a stronger relation than clusters further away from each other;

Cluster 1: Variation and attributes; Cluster 2: Variation and challenging, motivating, exciting, and adventurous; Cluster 3: Active games, and challenging, motivating, exciting, and adventurous; Cluster 4: Organized activities and affordable; Cluster 5: Clean, well maintained, attractive and suitable area; Cluster 6: Proximity; Cluster 7: Facilities, weather and safety; Cluster 8: Ambience and seated activities not encouraged; Cluster 9: Being forced to be active, being allowed to be active and challenging, motivating, exciting and adventurous;

Note that the two light grey clusters have disappeared, and one new cluster (not shown on the map) was created as a result of reallocation of statements: Cluster 10: Different target groups and presence of others (positive/negative).


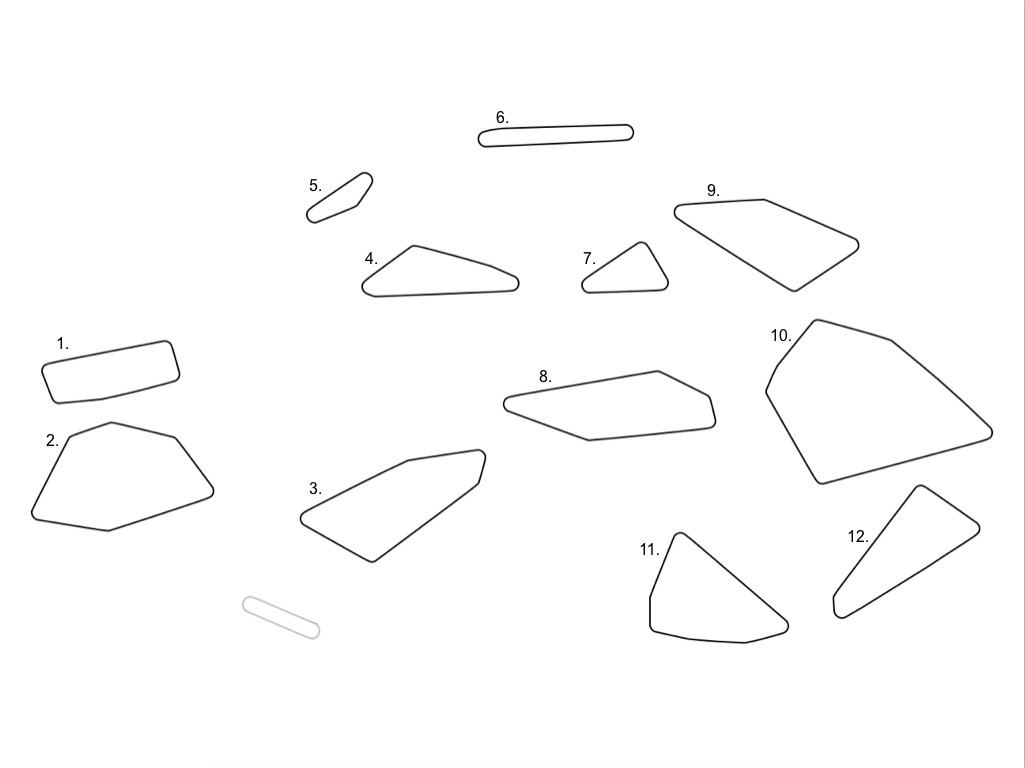


**Figure 3.** Concept map of year 4 of 6-year pre-university secondary education

Note that clusters closer together on the map have a stronger relation than clusters further away from each other;

Cluster 1: Well maintained and suitable area; Cluster 2: Clean, attractive and distraction (positive/negative); Cluster 3: Affordable and proximity; Cluster 4: facilities; Cluster 5: Safe; Cluster 6: Attributes; Cluster 7: Seated activities not encouraged; Cluster 8: Different target groups and it is the norm to be active; Cluster 9: Variation and challenging, motivating, exciting and adventurous; Cluster 10: Challenging, motivating, exciting and adventurous; Cluster 11: Ambience and presence of others (positive/negative); Cluster 12: Being allowed to be active and presence of others (positive/negative);

Note that the light grey cluster has disappeared as a result of reallocation of statements.


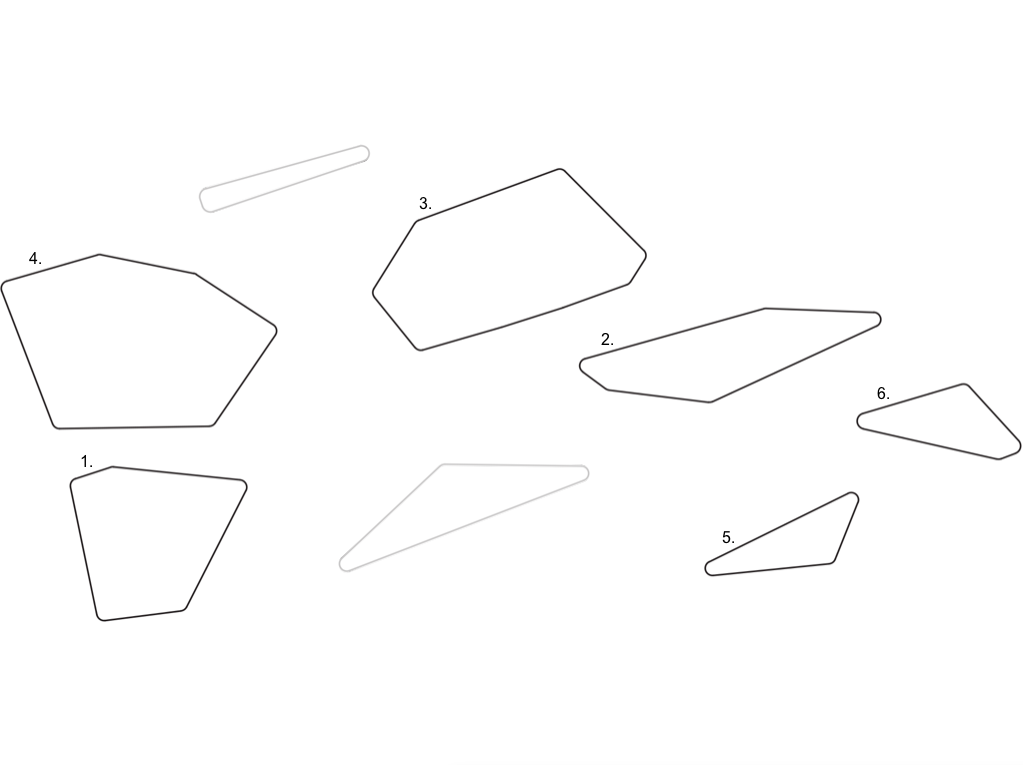
**Figure 4.** Concept map of year 4 of 4-year pre-vocational secondary education

Note that clusters closer together on the map have a stronger relation than clusters further away from each other;

Cluster 1: Ambience, presence of others (positive/negative), different target groups and challenging, motivating, exciting and adventurous; Cluster 2: Affordable and proximity; Cluster 3: Being forced to be active; Cluster 4: Variation, attributes and challenging, motivating, exciting and adventurous; Cluster 5: Safety and weather; Cluster 6: Clean, well maintained, attractive and suitable area;

Note that the two light grey clusters have disappeared, and one new cluster (not shown on the map) was created as a result of reallocation of statements: Cluster 7: Distraction (positive/negative).
