# Supplementary material for: An activity-friendly environment from the adolescent perspective: a concept mapping study
Source: Int J Behav Nutr Phys Act. 2018 Oct 16;15:99. doi: 10.1186/s12966-018-0733-x (PMC6192111; doi:10.1186/s12966-018-0733-x)
Supplement: Supplementary file 2 — Cluster compositions and importance ratings. Cluster compositions and average importance ratings of the underlying ideas. (DOCX 45 kb) [file 12966_2018_733_MOESM2_ESM.docx]

## Additional file 2: Cluster compositions and importance ratings

Table 1. Clusters, underlying ideas and importance ratings of year 2 of 6-year pre-university secondary education

|  | Clusters and statements | Importance |
| --- | --- | --- |
| 1. | **Variation, attributes and weather**  2. If there is a high swing  12. If there is a field where you can play/participate in a sport  13. If there is a free paddle boat park  19. If there is some kind of outdoor gym (equipment outside)  23. If there is a sports hall  25. If there are climbing walls  28. If there is a skate park  32. If there are many obstacles  34. If there are many activities in one place (high efficiency)  37. If there are sport attributes available  38. If there is a free run park  41. If there is an indoor playground for when the weather is bad  42. If there is a panna cage  46. If there are activities that you can do  47. If there is something in the neighborhood where you can do the activity, a lake for swimming or a tree to climb in, for example  48. Attributes available to be active with, like a parkour track, climbing rack, basketball net, or a trampoline  52. If there is a swimming pool  63. If there is an amusement park  69. If there are new sports that I don’t know yet/have never heard of  70. If you can combine different sport  71. Materials for strength training  76. If there is an athletics track  77. Racing bikes in an appropriate place  79. A go-kart track with karts  10. If there are running-friendly running tracks  27. If there is an island in a lake where you can go (e.g. only with a boat)  39. If there is a good combination of different sports fields  49. Attributes that look attractive  36. If the houses have gardens (or shared gardens/roof terraces)^a^  54. If the space is not flat and has many obstacles (e.g. hills) ^a^ | 2.50  3.90  2.36  3.36  3.00  3.10  2.55  3.18  4.30  4.40  2.82  3.36  2.42  4.30  3.73  4.30  3.10  2.80  3.55  3.10  2.82  2.18  2.73  3.27  3.00  2.40  3.55  3.36  1.70  3.64 |
| 2. | **Clean, well maintained, safety and distraction (positive/negative)**  1. If there are no cars  3. If there are no busy motorways  29. If there is no smoking  33. If there are no sweet machines  35. If there are no dogs allowed  43. If it is safe (e.g. no motorways in the area, or dangerous people)  62. If it doesn’t smell (like exhaust gases, factories)  68. If there is no access to the internet/Wi-Fi, then you won’t get distracted by your phone  64. If there are no distracting activities where it’s not necessary to be active  30. If it is well-kept and clean^a^ | 3.90  3.70  4.09  2.42  2.30  4.00  3.92  2.10  2.55  4.40 |
| 3. | **Attractive, seated activities not encouraged and suitable area**  7. If there is music with a fast rhythm  18. If there are not too many seating areas  21. If there is a fairly cold temperature  24. If there is lighting (evening)  45. If there is space to be active in (large and open)  55. If the space is outside  57. If there is a lot of green (nature) in the area  58. If it is light, so in the daytime  61. If there is a nice temperature (not too hot and not too cold)^a^  31. If there is a proper surface for all kinds of sports/kinds of activities (e.g. skating)^a^ | 2.20  2.17  1.80  3.50  4.64  4.20  3.73  2.91  3.00  4.20 |
| 4. | **Rewards and organized activities**  6. If activities are organized in playgrounds (competitions, tournaments)  80. A soccer tournament, of you win, you get money  59. If you get a present/rewards for being active, food for example^a^ | 2.50  1.70  1.82 |
| 5. | **Active games**  22. If there are no seated games available  51. If Virtual Reality (VR) is used  53. If you’re alone and can do the movements in front of a television (you get bored less quickly) | 2.30  2.00  1.90 |
| 6. | **Facilities**  14. If there are drinking water fountains in the area  50. If there is tap water to drink  60. If there is a place to store your stuff  67. If there is a community centre in the area  73. Clothes for sports^a^  40. If there is a place to relax, and have something to eat and drink^a^ | 4.50  4.45  3.80  1.64  1.90  3.83 |
| 7. | **Ambience, being allowed to be active and presence of others (positive/negative)**  4. If there are (nice) people who are also active  5. If there are not too many people  8. If there are no police officers (otherwise you will also climb trees, etc.)  11. If there are no strict rules about what you can and can not do  16. If there are not too many people watching you (e.g. in a forest)  20. If there are few elderly people (with regard to complaining about nuisance)  56. If there are no other people, then you’ll have more space  66. If there is supervision  26. If you can participate in a sport/exercise together  75. If there are trainers  81. It has to be fun for everyone  17. If there is a place that not too many people know about^a^ | 3.50  2.30  3.40  3.33  2.20  2.82  2.82  2.30  4.00  1.80  3.82  2.80 |
| 8. | **Affordable and proximity**^b^  72. If it is for free  65. If the area is central  9. If you can swim for free, for example | 4.00  3.30  2.82 |
| 9. | **Challenging, motivating, exciting and adventurous**^b^  78. That it is not very clear what you should do, that you have to think for yourself about the activities that you can do with what is available  15. If there is a playground for adults (more exciting and bigger playground equipment)  74. Child-friendly but in a mature way  44. When it is cold you want to move to get warm | 2.00  2.80  2.70  2.17 |

^a^ Indicates a statement is reallocated by researchers

^b^ Indicates a new cluster is created by researchers as a result of reallocation of statements

Table 2. Clusters, underlying ideas and importance ratings of year 2 of 4-year pre-vocational secondary education

|  | Clusters and statements | Importance |
| --- | --- | --- |
| 1. | **Variation and attributes**  21. If there are more mountain bike trails  22. If there are more grassy areas  23. If there is a beach or a park nearby, for beach volleyball for example  62. If there is more to do in the direct environment  67. If there are enough hiding places  71. If there are big trees you can climb  81. If there are walls or trees you can climb in or over  1.If there are more possibilities (playground equipment) to be active in the playground, for example cool slides, a cable car, ball pit, karting track^a^  17. If you can do several sports/activities because there are various sports fields, for example a soccer pitch, basketball court, athletics track^a^  63. If there are some goals^a^  72. If there is play and/or sports equipment^a^  77. Fitness equipment outside, in the park for example^a^  47. Fitness equipment in shopping centres or at home^a^ | 2.62  3.18  3.38  3.23  2.95  2.97  2.87  3.38  3.74  3.15  3.36  3.28  2.95 |
| 2. | **Variation and challenging, motivating, exciting and adventurous**  3. If being active in a certain area continues to be fun for a long time  5. If there is the opportunity to try out all kinds of (original) sports in the area, for example miniature golf, yoga, swimming  14. If the area is more challenging due to the availability of playground equipment  15. If you can also do activities in the dark, for example, hide and seek, glow in the dark  18. If there is a place for role-playing games, for example two castles across from each other, or playing mothers and fathers  25. An obstacle course or storm track where you can run  27. If you can do various activities in an indoor environment for a couple of hours, trampoline jumping for example  31. If there is something to do that you have never done before (original playground equipment)  35. If there is a special activity (e.g. storm track)  64.If it looks challenging (encouragement through the environment)  73. The possibility to compete in contests  16. If there are more fun things to do on the way to school^a^  97. If there are things to photograph^a^ | 4.18  3.74  3.92  3.13  2.51  3.28  3.15  3.67  3.38  3.56  3.31  2.96  2.36 |
| 3. | **Active games and challenging, motivating, exciting and adventurous**  7. More virtual opportunities to be able to be active  9. Activities that give you a kick, sports that you do not often do, many different activities available  10. The ability to visualise your improvements, for example with a timer  54. If you are able to figure out how something works by yourself  79. If technology is used in sports (e.g. a dance mat)  95. If there are exciting activities to do (e.g. paintball), adrenaline  43. If there is a soccer wall that instructs you where to shoot and measures how hard you shoot^a^  44. If activity in the environment contains a game component^a^  88. If more active games (e.g. on your phone) are made available (e.g. just dance)^a^ | 3.05  3.58  3.18  3.21  3.33  3.74  2.49  3.21  3.13 |
| 4. | **Organized activities and affordable**  11. If activities are organized, for example morning warming-up exercises  74. If you have to search for something, for example Easter eggs  92. If it is for free or cheap, for example, less expensive sports clubs  94. Fun activities for little money, laser games and go-karting is expensive  58. If treasure hunts, sports days, sponsor runs, or competitions are organized, for example^a^  68. If there is an explanation of the activities available^a^ | 2.69  2.31  3.54  3.90  3.08  2.74 |
| 5. | **Clean, well maintained, attractive and suitable area**  12. If there is a clean public toilet  20. If it is outside  28. Where there is enough oxygen and fresh air  30. If something is removed, it must be replaced  34. It should look clean and well-kept  39. If there is a paved surface, for skating or running for example  40. If it looks attractive (colourful, not just grey but other colours)  41. If there are no bushes or ditches around (ball in the ditch)  45. If there are not too many obstacles (e.g. trees on the soccer pitch)  59. If the environment is well maintained (e.g. that the grass is not too soggy)  61. If there is a lot of nature, forest or flowers and plants in bloom  65. If the fences are made of wood, nature is incorporated in the playground  66. An activity at a nice location, at the beach or surrounded by green/nature, for example  87. If there is better lighting so that you can be active in the evening  26. If there is space you can be active in and perform sports, a spacious/big sports field^a^  60. If there are fields with artificial grass, instead of brick^a^  49. Playground equipment that is solid and not in need of repair, equipment is sometimes vandalised^a^  29. If it is not too hot^a^ | 3.67  3.00  3.97  3.41  4.03  2.95  3.85  2.74  2.59  3.76  3.13  2.18  3.13  3.41  3.85  3.21  4.21  2.54 |
| 6. | **Proximity**  36. If the roads are better maintained  70. If it is nearby and easily accessible  42. If it is at a good location^a^ | 2.75  3.18  3.85 |
| 7. | **Facilities, weather and safety**  6. If there are no groups of young people loitering  33. If parents give you more freedom, because the environment is safe  46. If there is a shopping centre in the area (you’ll walk a lot)  85. If there is food available in the area  48. If there are more drinking water fountains^a^  50. If there is no motorway in the area^a^  53. Indoor sports, all the year round^a^  78. An information desk where you can borrow equipment in exchange for a deposit^a^  84. If there is a possibility to go inside (in case of rain or when it’s cold)^a^  91. If there is cold water to play with or swim in when it’s hot^a^ | 2.67  3.31  2.79  2.74  3.47  2.40  2.87  2.95  3.43  2.90 |
| 8. | **Ambience and seated activities not encouraged**  52. If there are no screens that make you want to sit down  55.If there are more stairs, no lifts or only with an access pass  75. If there are many people present, cosy  82. If there are no lifts or escalators  83. If there are not too many people  89. If there are no lazy people around (it is demotivating)  93. If there are little or no chairs, benches, etc.^a^ | 2.69  2.51  3.10  2.85  2.41  2.00  2.51 |
| 9. | **Being forced to be active, being allowed to be active, distraction (positive/negative), rewards and challenging, motivating, exciting and adventurous**  2. If there is more space in the classroom to be active  37. If you are allowed to do anything you want to do (regardless of age, height)  51. If you are forced to be active (no bus to school)  56. Rewards: food, scoring points (Pokémon app)  69. Posters or ads that motivate you to participate in sports  76. If I’m allowed to be active (e.g. in school)  86. If an app stimulates people to go outside  90. Being motivated by a person  57. Snack bar/unhealthy foods only accessible by bike^a^  8. You are forced to do an activity before performing a daily activity, e.g. running to the toilet before using it^a^  80. If there is music in the background (e.g. for distraction)^a^ | 3.67  3.89  2.38  2.56  2.56  3.51  3.10  2.69  2.41  2.23  3.23 |
| 10. | **Different target groups and presence of others (positive/negative)**^b^  24. If the playgrounds are focused on age  32. If the environment is aimed at the youth (e.g. graffiti)  96. If there is the opportunity to participate in a sport/be active with other people  4. If there is something to do for the parents too  19. If there are separate pitches for different ages (less crowded per pitch)  13. If you maintain the school garden at school together | 3.28  2.95  3.54  2.83  3.23  2.05 |

^a^ Indicates a statement is reallocated by researchers

^b^ Indicates a new cluster is created by researchers as a result of reallocation of statements

## Table 3. Clusters, underlying ideas and importance ratings of year 4 of 6-year pre-university secondary education

|  | Clusters and statements | Importance |
| --- | --- | --- |
| 1. | **Well maintained and suitable area**  1.If there is a suitable floor, in many cases no brick floor (not so hard, safe)  2. If there is a good surface for participating in a sport (depending on the sport)  4. If there is enough space  5. If there is enough space to be active in (individually)  22. If there are no breakable objects in the area  23. If it is well-kept (no lawn without grass/ broken climbing frames)  25. If the area is well-kept (not old/broken)  96. If the surface is soft and firm (functional and safe)  6. If there is enough space in traffic (cycling, walking)^a^  19. If there are not too many obstacles in the area (e.g. waste bin, bench)^a^  21. If there are no fences that prevent activities, no blockages^a^  11. If the environment has the right temperature (e.g. a gym that is ice cold or too hot is not pleasant)^a^  64. If there is fresh air (clean and temperature)^a^  61. If the place is functional for the activity that you want to do^a^ | 3.54  3.95  4.29  4.16  2.58  3.92  4.05  3.24  2.66  2.74  2.50  3.34  3.89  3.92 |
| 2. | **Clean, weather, attractive and distraction (positive/negative)**  26. If the area is clean (no waste/dirt/sweat on the equipment)  27. If there is no dog poo  32. If the room is well-lit (preferably sunlight instead of cold white light)  33. If the area is well-lit (you should be able to see what you are doing)  38. If the sun is shining, if it is not raining  45. If it does not look boring (e.g. not the colour grey)  46. If the area is green (plants/forest)  47. If the area is simple/quiet (no distracting posters/ads)  48. If it looks nice or attractive  49. If the environment is modern  50. It it is colourful  65. If the area is quiet (e.g. no distraction of a busy road)^a^  90. If the area is not too quiet^a^  66. If there is music^a^  67. If there is a distraction, so you don’t notice you are tired (e.g. music)^a^  85. If there are no dogs that distract you (then you’ll want to pet them)^a^ | 3.58  2.82  2.68  3.50  2.39  2.47  2.63  2.03  2.89  2.53  1.95  2.89  1.71  2.84  3.08  1.53 |
| 3. | **Affordable and proximity**  34. If it is near your home (if it is too far, you’ll go less often)  35. If the place is accessible  36. If the place is centrally located (accessible to all)  82. If being active in the environment is always possible  86.If there is a clear route indicated (signs)  81. If you don’t have to pay for the space (financially)^a^ | 3.55  3.89  3.45  3.67  2.39  3.58 |
| 4. | **Facilities and weather**  71. If water and food are available (drinks or energy bars)  72. If there are facilities to freshen up (showers)  73. If there is a place to relax  74. If there are facilities (toilet, food)  37. If there is a covered area to shelter from the rain^a^  98. If there is the possibility to participate in a sport in sportswear^a^  87. If the possibility to be active is not obstructed (availability of a bicycle parking garage)^a^  39. If it’s a place where you are not dependent on the weather^a^ | 3.13  3.08  3.29  3.58  3.05  3.18  2.74  3.11 |
| 5. | **Safety**  13. If there is safe sports equipment available  20. If you can easily access attributes (no briar patches/ditches)  24. If attributes (e.g. climbing frame) are made of strong material  12. If it is not a dangerous place (you can get hurt)^a^ | 3.73  2.92  3.32  3.82 |
| 6. | **Attributes**  7. If there are attributes available (fixed/loose) (fitness equipment/a football)  8. If there are moving attributes available (swing/trampoline)  9. If there are technological attributes available, an odometer for example (feedback)  10. If there are things that you can do something with (e.g. in a gym), or there is music, so you can dance | 3.71  2.95  2.71  3.50 |
| 7. | **Seated activities not encouraged**  79. If there are no chairs, then you will stay seated (provokes passive behavior)  80. If there are few objects that provoke seated behavior | 1.95  2.05 |
| 8. | **Different target groups and it is the norm to be active**  29. If it is accessible for every level  31. If it is accessible to all (also for people with walking difficulties)  63. If the purpose of the area is to be active  88. If active transport is faster than passive transport  89. If the environment forces you to be active (e.g. no cars, only a bike)  60. If the name of the ‘building’ makes being active normal (in an office you won’t be active; the norm)^a^  30. If it is possible to be active at different levels (separately)^a^  97. If it is a kind of obligation to go outside^a^  62. If it is normal to be active there (that it is not conspicuous)^a^  28. If it is suitable for every age (playground/running track)^a^ | 3.08  2.53  3.45  2.50  2.42  1.47  3.32  1.87  3.08  2.71 |
| 9. | **Variation and challenging, motivating, exciting, and adventurous**  15. If you are physically challenged (enough weights for bench press, to be able to improve step by step)  18. If there is a game element  52. If it is directed at various sports (that there are fun activities for different kinds of people)  53. If there is a variety of opportunities to be active close to each other  54. If variation is possible  84. If the area has activities that you can only do there (special)  51. If there is variation in play equipment and quantities (enough to do for a longer period)^a^ | 3.82  2.82  3.08  3.16  3.59  3.00  3.24 |
| 10. | **Rewards and challenging, motivating, exciting, and adventurous**  16. If there is room for competition (matches)  17. If your creativity is tested  68. If it gives you a good feeling, that you can win something (reward)  70. If you get something in return for being active  76. If there are people who can help you (challenge and motivate you, matching your level and interest)  78. If you can improve yourself  83. If the environment encourages you to do something you have not done before (new)  69. If being active has a purpose (charging your telephone)^a^  75. If you are supported by an external person (so you do it the right way/no wrong exercises)^a^  77. If there is information about the effects of being active^a^ | 2.97  2.55  3.11  2.66  2.95  3.89  3.26  2.45  2.84  1.84 |
| 11. | **Ambience and presence of others (positive/negative)**  3. If there are not too many people in the space  14. If both the furnishing and the people are safe (socially; loitering)  42. If you can not cause a nuisance (that you do not bother anyone, e.g. noise)  91. If the ambience is nice  93. If there is enough privacy, no non-sporting people watching others participating in a sport (if you are sweating/have a red face)  94. If there are no people who are shouting/scolding in a negative way  95. If it is a private area (without other people) | 2.92  3.39  2.63  3.79  2.76  2.95  2.42 |
| 12. | **Being allowed to be active and presence of others (positive/negative)**  40. If you are free to do whatever you want to do (no one tells you what to do)  41. If there are no people who don’t want you to be active there (people who prick holes in your ball)  55. If the area is already being used by active/sporting people (it sounds attractive to join in)  56. If the environment makes being active/participating in a sport together possible (with friends/team sports)  57. If it encourages you to be active together  58. If it causes social interaction  59. If you can play there with people of different ages (play together with little children)  92. If you don’t run into people you know, so that you have to be sociable  44. If you are free to be active (no restrictive rules to prevent activity)^a^  43. If you’re allowed to be somewhere (e.g. fitness hall, soccer pitch)^a^ | 3.82  3.63  2.82  3.53  3.05  2.74  1.82  2.13  3.89  3.21 |

^a^ Indicates a statement is reallocated by researchers

Table 4. Clusters, underlying ideas and importance ratings of year 4 of 4-year pre-vocational secondary education

|  | Clusters and statements | Importance |
| --- | --- | --- |
| 1. | **Ambience, being allowed to be active, presence of others (positive/negative), different target groups, and challenging, motivating, exciting and adventurous**  1. If it is cosy  2. If it is for a specific group (small group)  6. If I’m away from stress (abroad)  7. If I’m with friends  11. If there are a lot of peers  15. If there is excitement  16. If there is adventure  22. If there is something you can’t do in the Netherlands but can abroad, you will try it faster  41. If there are a lot of people participating in sports  44. If there are many people with whom you can have fun  49. If there are no rules and you can do whatever you want  4. If it is child-friendly (non-smoking/-drinking)^a^  47. Accessible for all^a^ | 4.63  2.25  3.50  4.75  3.75  3.63  3.50  3.63  3.38  4.00  3.13  3.88  4.25 |
| 2. | **Affordable and proximity**  3. If there are decent roads leading to it  29. If it is near your home environment  23. If it is cheaper (e.g. abroad)^a^  24. Depending on space rental and availability^a^  58. If the sport does not cost any money (for free)^a^ | 3.25  3.50  3.00  2.63  3.75 |
| 3. | **Being forced to be active**  8. If I have to be active (going to the supermarket, for example)  51. Many lifts in a building make you less active  50. If a building or a home has many stairs^a^ | 2.88  3.13  2.13 |
| 4. | **Variation, attributes, facilities, and challenging, motivating, exciting and adventurous**  9. If there are shops (you can go shopping)  12. If there is a trainer (being pushed to carry on)  13. If it is motivating (e.g. spectators)  14. If there are fun activities  17. If it is challenging (if you have never done something before)  18. If you can zip down a zip line  19. If you can swim  20. If there are really high swings  21. If you can go snorkeling  26. If there are playgrounds and restaurants  28. If you can be scouted  31. If there is an ice skating rink  32. If there are trampolines  33. If there is a children’s farm (together with nephews and nieces)  52. If there is a sports club  53. If there is someone in the area that puts you to work (if there is supervision)  56. If there is water, to swim in for example^a^  30. If there is a sea^a^  57. If there is drinking water available^a^  27. If you know there is something to do (advertising)^a^  43. If there are bicycle lanes^a^  25. If there are different soccer pitches, many different sports available in one area^a^  39. If there are (sports) attributes available^a^  46. Many gyms^a^ | 2.63  2.88  3.25  4.00  3.38  2.50  2.88  1.50  1.63  2.88  3.25  1.75  1.75  2.00  3.25  2.75  3.00  2.13  4.13  2.00  3.13  4.13  4.00  2.75 |
| 5. | **Safety and weather**  5. If the weather is nice  38.The weather (nice/bad weather)  59. If you feel safe, if there are no groups of youths loitering for example  61. If it is quiet | 4.25  4.13  4.13  3.88 |
| 6. | **Clean, well maintained, attractive and suitable area**  10. If there is nature  34. If there are no motorways in the area  35. If it is free of cars and scooters (less CO2)  37. Clean, not dirty, no trash  60. If it is outside  40. Nice and fresh-smelling air in the room^a^  36. Open, a lot of space (like a soccer pitch)^a^  42. If the environment is well-kept (no pot holes or tree roots)^a^ | 3.25  3.13  2.75  4.63  3.50  4.25  4.13  4.50 |
| 7. | **Distraction (positive/negative)**^b^  55. If you are with a small group, you’ll get less distracted  48. If you are not in the home environment, couch/bed make me less active  54. If there are no things around that distract you (a computer for example)  45. Little noise (machines or cars) | 2.88  2.88  2.63  3.50 |

^a^ Indicates a statement is reallocated by researchers

^b^ Indicates a new cluster is created by researchers as a result of reallocation of statements
